# Experiences of midwifery students and graduates in Somalia: evidence from qualitative data

**DOI:** 10.3389/fgwh.2024.1480914

**Published:** 2025-01-17

**Authors:** Hawa Abdullahi, Asia Mohamed Mohamud, Maryan Abdulkadir Ahmed, Mohamed Ahmed Omar, Abdirisak A. Dalmar, Hannah Tappis, Shatha Elnakib

**Affiliations:** ^1^ Research, Somali Research and Development Institute, Mogadishu, Somalia; ^2^ Center for Humanitarian Health, Johns Hopkins Bloomberg School of Public Health, Baltimore, MD, United States

**Keywords:** midwifery experience, graduates and students, conflict-affected settings, Somalia, qualitative research, cohort study

## Abstract

**Introduction:**

Midwives play an important role in maternal and newborn care, yet are in short supply globally. The shortage in midwives is particularly acute in sub-Saharan African countries, many of which are conflict-affected. Midwives face many challenges that impact their workforce participation and retention, and these challenges are often compounded in conflict settings. Somalia ranks among the countries with the highest maternal mortality rate, with an estimated shortage of 20,000 midwives compared to the WHO recommended standard. Yet, limited research has explored the lived experience of midwives in conflict. This qualitative study seeks to explore the experiences, plans, and aspirations of midwifery students and recent graduates from nine midwifery schools in Somalia and to understand how the safety and security situation impacts their educational experience and willingness to join the profession.

**Methods:**

This is a sub-study embedded within a broader prospective multi-cohort study of midwifery students and early career midwives conducted in 2023 and which will continue until 2025. We invited graduates and students in their final year from eight schools in Mogadishu and one school in Galgadud to participate in the study. This study draws on findings from five focus group discussions conducted with midwifery students and graduates who were included in the parent cohort study, for a total sample size of 33 students and graduates. We conducted thematic analysis using a combination of inductive and deductive coding.

**Results:**

Our data illustrates powerful motivation among midwifery students and graduates to join the profession. Motivation was mostly intrinsic, with participants describing their desire to help the mother-baby dyad and to reduce maternal mortality in their communities as the predominant driving force for joining the profession. Yet, participants cited several barriers to entering the workforce, including harmful gender norms that prioritize women's domestic roles over professional aspirations and societal mistrust toward young midwives. Participants stated that their age and gender undermined them professionally and noted the community's preference for older midwives and traditional birth attendants. Safety and security significantly impacted participants' acceptance of job placement in remote areas and students' ability to attend school regularly. In terms of perceived preparedness, graduates felt well prepared for their role but identified gaps in abortion care, neonatal resuscitation, and usage of basic ultrasound.

**Conclusion:**

The challenges faced by midwifery students and newly graduated midwives have direct implications for the health outcomes of mothers and infants in conflict-affected settings. The recurring themes of inadequate training, security risks, and restrictive gender norms require holistic and systematic interventions that address these issues in order to improve motivation and workforce participation among this important health cadre.

## Introduction

Midwives play a critical role across the maternal and newborn health (MNH) care continuum. Evidence demonstrates that midwives educated and regulated to international standards could help prevent more than 80% of all maternal, stillbirth, and neonatal deaths (1). Yet according to the most recent State of the World's Midwifery, there is a severe shortage of midwives, with an estimated shortfall of 900,000 (2).

Despite the important role played by this health cadre, the profession is beset by a range of challenges that have led to high rates of turnover and workforce attrition (3). Historically and across settings, midwives have had to wrestle with perceived low professional status in the community, high workload, violence in the workplace, insufficient resources, and other work-related stressors, leading to burnout and reduced job satisfaction (3, 4).

The shortage of midwives is particularly problematic in Sub-Saharan African countries, and especially those that are crisis-affected, as those settings tend to report high maternal mortality and high fertility rates coupled with health personnel shortages. Somalia has the highest maternal mortality rate, estimated at 621/100,000, and the number of health professionals across all cadres falls short of the World Health Organization's (WHO) recommended standards, resulting in an estimated shortage of 20,000 midwives (5).

There are few studies investigating the experiences of early career midwives in humanitarian settings who are new to the profession and must work in complex and demanding contexts characterized by pervasive insecurity. Evidence from both stable and humanitarian settings indicates that attrition from midwifery typically occurs in the early career period, defined as the first five years of practice (6). After the collapse of the central government in 1991, the country's health infrastructure was largely destroyed during the civil war, with many health centers looted or taken over by armed militias and internally displaced people. This resulted in the loss of medical professionals and the annihilation of training institutes, which led to a severe shortage of qualified health workers (7). In order to address the shortage of skilled midwives in Somalia, the country has implemented several reforms. For example, in 2012, the Ministry of Health (MoH) of the Somalia Federal Government and the United Nations Population Fund (UNFPA) established four midwifery training institutes across the country. Moreover, the national reproductive health strategy in 2010–2015 focused on three priorities: birth spacing, safe delivery, addressing harmful practices like female genital mutilation, and galvanized commitment among all partners on improving midwifery workforce development (8).

Additionally, private universities began offering direct-entry midwifery programs. Also, many private universities have started offering nursing and other healthcare programs. Three types of midwifery education programs are currently provided in Somalia: (a) integrated nursing and midwifery, (b) a four-year bachelor's degree midwifery program, and (c) a three-year midwifery diploma program.

A limited number of studies have been conducted on midwifery in Somalia, despite the critical role played by this cadre in providing lifesaving maternal health services. The few that have been undertaken indicate that midwives face complex challenges including lack of training, high workload, and lack of defined work scope, which have impacted their provision of quality of care (9, 10). Tensions with other health professionals and unclear professional responsibilities also hinder their ability to provide midwifery-led care (9, 10). Moreover, there is a notable dearth of studies on the experiences of midwifery students and the educational and safety challenges they face.

This study aims to elevate the voices and experiences of early career midwives working in conflict settings and to inform interventions grounded in participants' experiences that will increase the retention and motivation of this vital health cadre. Specifically, this paper aims to explore the experiences, plans, and aspirations of midwifery students and recent graduates and to understand how the safety and security situation impacts their educational experience and willingness to join the midwifery workforce.

## Methods

### Study design and setting

This qualitative study is nested in a broader prospective multi-cohort study of midwifery students and early career midwives that was initiated in 2023 and is planned to continue until 2025. The overall study seeks to generate evidence on factors affecting midwifery students' and early career midwives' aspirations, workforce participation, and retention. In this study, we draw from qualitative data that we collected from students and graduates.

The study was conducted in the Bendir and Galgaduud regions. Benadir region (Mogadishu) is the capital and largest city of Somalia and host to the largest protracted internally displaced population. Mogadishu has experienced significant challenges related to insecurity and violence stemming from the ongoing conflicts between the government and Alshabaab. Whereas Galguduud is an administrative region of Galmudug State in central Somalia, near the border with Ethiopia. Conflicts between clans and clashes with armed groups persist in Galgaduud. The security situation across Somalia is unpredictable. The country is also experiencing food insecurity and climate shocks, which complicate humanitarian access.

We mapped out the schools that provide direct entry midwifery programs from a total of 16 schools in Mogadishu. We purposefully selected and invited all students in their final year from seven private schools and the only one community midwifery school in Mogadishu, as well as from the one midwifery school that exists in Galgaduud. Those willing to participate were then administered a survey and informed that they will be interviewed several times over a follow-up period of 3 years and that a subset of participants may be contacted to participate in in-depth interviews or focus group discussions. Of those who were enrolled in the cohort study and administered the baseline, we randomly selected six groups of 6–8 midwifery students and graduates to participate in focus group discussions (FGDs), the findings of which are presented in this manuscript. In total, we conducted the following:

1. Two FGDs with midwifery students in Mogadishu;
2. Two FGDs with recent midwifery graduates in Mogadishu; and
3. One FGD with recent community midwifery graduates in Galgadud.

### Participants

We sought permission from the deans of the schools to visit them and enroll interested students in their last year of the program. The students were informed in advance about our team's visits, and those willing to participate provided oral consent and enrolled in the study. Participants were selected based on the following inclusion criteria: We selected (1) students currently enrolled in the midwifery program from the underlying cohort of students who were enrolled in the cohort study, or (2) students willing to provide verbal consent for in-person interviews, and (3) midwifery graduates from the underlying cohort of graduates enrolled in the longitudinal study. The study team collected their contact information for follow-up during their enrollment in the longitudinal study. We then randomly selected two students or graduates from each school to ensure representation across all schools. We contacted the selected participant to confirm their availability and willingness to participate in the Focus Group Discussions (FGDs). We utilized FGDs because we were interested in gauging group experiences and perceptions of the midwifery program and identifying commonalities and differences across students' and early career midwives' experiences. We administered two FGDs for students and three for the graduates, with a minimum of six and a maximum of eight participants interviewed per FGD, for a total sample size of 33 midwifery students and graduates. 'Students' refers to participants in their final year of study, while “graduates” denotes those who had completed their studies at the time of data collection. Although this study did not explicitly compare these two groups, as the primary focus was to capture a comprehensive understanding of midwifery in Somalia, notable differences between them are highlighted where relevant.

### Data collection

Initially, baseline cohort recruitment was carried out for students in their final year of study. These students were administered a survey designed to collect information about their academic experience and enroll them into the cohort study for future follow-up. This article does not feature any data from the survey.

Following the survey, we conducted FGDs to gain a deeper understanding of the experience of midwifery students and graduates. FGDs with graduates aimed to understand their intentions regarding practicing midwifery, their job search experiences, their job expectations, and how conflict impacts their experiences and desire to join the workforce. FGDs with students sought to gain deeper insights into their experiences within the program, the extent to which they feel their education is preparing them to become midwives, and the challenges they face, particularly those arising from conflict and insecurity.

Three data collectors, two males and one female with backgrounds in health and social sciences and with three to five years of experience in data collection and research, were trained over three days in human subject research and the data collection tool. Each FGD was conducted by two data collectors, with one female data collector facilitating the discussion and obtaining consent from participants, while the other took notes, documented verbal and non-verbal cues, and collected participants’ sociodemographic data. To minimize potential for bias, the female data collector was assigned as the facilitator, and the males were assigned the roles of notetakers.

All data collection was conducted in Somali and later transcribed directly into English for analysis.

The FGDs occurred in the Somalia Research and Development Institute Offices in Mogadishu and Diriye Halls in Galgaduud from December 2023 to January 2024. This allowed participants to speak freely and ensured they had visual and auditory privacy.

### Data analysis

All FGDs were audio recorded, transcribed, and imported into the qualitative data management software Dedoose. AM and HA coded all the data. We used a codebook developed deductively based on the study objectives and added codes inductively when new codes emerged. Deductive codes reflected our interest in understanding challenges experienced by students in relation to their academic program, safety and security of students and graduates, perception of preparedness to work as midwives, and viewpoints about how the community perceives the midwifery profession and how supported students and graduates feel. Inductive codes emerged around intrinsic motivation and suggestions for program improvement. Our analysis involved memos and extensive discussions with the study team. In March 2024, an analysis workshop was organized in Somalia to review and analyze the coded data and to discuss preliminary findings. The workshop was attended by four of the study authors (three in-country and the last author, who is based in the United States). The team went over excerpts and discussed patterns emerging from the data. The discussions helped establish the credibility and accuracy of the findings and ensured that they resonated with the in-country team that collected the data.

### Ethical considerations

The study was approved by Institutional Review Boards at Johns Hopkins Bloomberg School of Public Health (IRB00024146) and the Somali Research and Development Institute (SORDI-EA02 52).

## Results

The study presents the perspectives of midwifery students and graduates. A total of 33 participants were interviewed, including 13 students and 20 graduates, all of whom were female. The participants' ages ranged from 19 to 26 years, and none were employed during the time of the interviews.

In the following section, we outline themes that emerged from the analysis, including motivation for joining the midwifery profession, midwives' perspectives on how they are perceived by the community, the safety and security situation and its impact on participants, and perceptions of preparedness.

### Motivation for joining the profession

Motivation to become a midwife was mostly intrinsic, with participants universally explaining their determination to practice midwifery as stemming from a desire to care for the mother-newborn dyad. Many cited the country's high maternal mortality ratio and their desire to help reduce it in their surroundings as a motivating factor. Extrinsic motivators, such as salaries, recognition, or prestige, were rarely mentioned, as participants noted that the midwifery profession is low-paid and not highly regarded. One participant shared an anecdote that motivated her to join the profession after she helped deliver a woman in a village:

“Once, when I was a child, I visited my father, who lived in a village. A mother sitting under a tree called me over. When I approached, I saw that she had given birth under the tree and was asking for help to cut the cord. I used a stone to cut the cord, while the baby lay in the sun. The mother had only a small cloth to cover herself. I helped her clean up the mess, gave her some water, and she went home. Now, as I reflect on that incident, I realize that with the knowledge I possess today, I could have assisted her more effectively by providing her with water, covering the child, and locating a cool spot. Every time I recall that incident, I am overcome with pain. (Recent graduate, FGD, Galgaduud)

Moreover, two participants two participants mentioned religion as an inspiration for choosing the profession. “The Quran says that saving one person saves humanity. *That is my inspiration. I am not seeking validation. I will help every pregnant mother I encounter, regardless of whether they have anything to offer. (Recent graduate, FGD, Galgaduud).* Another participant shared her happiness when a person she helped at work prayed for her: “*A mother's prayer is a joy. I feel the excitement when I help a pregnant mother, show sympathy, and deliver her baby. She will pray for you, and that will make you happy. These mothers’ prayers will help you succeed in life.” (Recent graduate, FGD, Galgaduud).*

Most of the participants were not only motivated by the desire to help women and children but also were anxious about the dominance TBAs had in the community. They personally witnessed the mismanagement of health conditions by TBAs due to their lack of proper training. Many participants quickly shared their mothers' or siblings' negative experiences with TBAs, attributing their desire to practice midwifery to the popularity of TBAs.

“I was in secondary school when my sister, who lived in a village in the Lower Shebelle region, got married and became pregnant. In her last trimester, she developed eclampsia and experienced dizziness, agitation, and blurred vision. The family believed she was possessed by a demon and began beating her. After a while, she moved to our house, where the family beat her again. Eventually, they recognized the symptoms and rushed her to the hospital, but she died before delivering the baby. That incident motivated me to study midwifery.” (Recent graduate, FGD, Mogadishu)

I am from a village where traditional midwives are common. My sister has an android pelvis, making normal delivery impossible. A traditional midwife assisted her at home, and she delivered a baby with an abnormally long head, who later died. After helping her, I was inspired to study this field in order to assist more mothers. (Recent graduate, FGD, Mogadishu)

Participants acknowledged the common societal expectations that limited women's social roles to the domestic sphere, but they saw midwifery as a means of empowerment and described the need to challenge traditional gender norms to achieve their goals of becoming midwives. Several participants felt like they developed ambitions or motivation after joining the midwifery program or volunteering as midwives.

“When I started midwifery, I became very ambitious. Moreover, when I began practicing and saved a mother and her child, I became filled with happiness.” (Midwifery student, FGD, Mogadishu)

“One day, I was told, ‘why are you learning this? Why don't you get married? Other people your age are already married.'“ I am determined to study to be able to do things tomorrow for my future and children, and I’m always sick of being told to get married. (Midwifery student, FGD, Mogadishu)

“If you study and possess the skills and knowledge to conduct procedures, you can work in health centers or establish your own ward to support mothers. What excites me is witnessing a mother who gave birth with my hands walking with her child after years.” I feel joyful and proud. I didn't just learn this to receive income; I learned this to enrich my mind and to be able to help the community.” (Recent graduate, FGD, Galgaduud)

### Community perceptions of midwives

Participants noted that their communities predominantly held negative views about young midwives. First, participants expressed concern about the preference for TBAs who were viewed as possessing more experience because they are generally older than early career midwives who are young and thus seen as lacking experience in delivering babies. In Somali communities, participants explained, there is a normative understanding of the midwife as an older woman. Participants reported experiencing distrust, disrespect, and belligerence at work due to the community members' lack of trust in their abilities. Instead, the community prefers TBAs for normal childbirth and doctors for complicated surgeries. Also, the community views midwifery as a female profession. Male midwives face challenges and rejection from the community, whereas male obstetric doctors are accepted.

“Some communities do not support young, educated midwives. They believe that only experienced TBAs can perform this job, and sometimes they do not trust us.” (Midwifery graduate, FGD, Mogadishu)

Secondly, participants reported widespread devaluation of the midwifery profession in their communities, with many sharing that family or community members believe that midwifery is unnecessary, easy, and lacks professional status. This affected the morale and motivation of participants, particularly those whose families discouraged them from pursuing midwifery. Some participants shared that their families perceived midwifery as a low-paying profession in comparison to other healthcare fields, and they believed that studying for four years to perform tasks that traditional birth attendants (TBAs) perform without formal training was a waste of time. Participants noted the lack of community awareness of the important role midwives play in saving mothers and babies and their incorrect belief that delivering a baby is a simple task of grabbing the baby from the mother.

My mother told me, “You are studying for four years to learn how to deliver a baby, but I gave birth to all of my children at home with the help of a TBA, except for one.” That makes no sense.” (Midwifery student, FGD, Mogadishu)

Thirdly, the community's perception of women's employment affects young, qualified midwives. Many people believe that girls should prioritize marriage and household work over pursuing a career, so young women are expected to focus on housework instead of becoming midwives. This societal pressure, in addition to the scarcity of jobs in safe areas, discourages many midwives from actively pursuing employment.

Most of the time, we are told to get married. Huge pressure is put on us when we are not ready for it, and they believe that an educated girl will end up in the kitchen.” (Midwifery student, FGD, Mogadishu)

However, a subset of participants shared that some community members have positive perceptions of midwifery and appreciate midwives' role in saving mothers and babies. Not only so, but they also see midwifery as a woman-friendly job that can be pursued from home and in the community, allowing women to fulfill their domestic duties. Despite the predominantly negative views espoused by community members, young midwives still expressed enthusiasm about pursuing their profession and helping mothers and babies.

Some community members encourage you to work in this field, and some discourage you, but that does not affect our decision. Some mothers, if you support them, show them kindness, and deliver from them; they praise you and encourage you to keep working, motivating you.”(Recent midwifery graduate, FGD, Mogadishu)

### Safety and security

A key element of the discussions centered around the impact of insecurity on students's learning and professional experiences. Participants reported the stress and anxiety their family members feel while their girls are away, especially when there is news of insecurity.

‘’ You might occasionally be required to work night shifts in remote locations such as “Weydow,” where there are numerous security concerns. If my mother heard a bomb blast or any other incident, she would become extremely stressed and her blood pressure would spike. (Midwifery student, FGD, Mogadishu)

“When it comes to safety, our area is plagued by numerous thieves on the roads.” Once, I got robbed by thieves, and they took my smartphone. In fact, I was robbed three times on that particular road. (Midwifery student, FGD, Mogadishu)

“I have grown up in this environment, and I have adapted to it. We don't know if we will go back home safely. When I am away from home, my parents always call to check on me. (Midwifery student, FGD, Mogadishu)

Students cited traffic jams and road closures that occurred because of insecurity as hindering school attendance. When deployed to the outskirts of the city or rural areas, students cited the risk of facing violence, especially gender-based violence.

“What's worse than that is that since we are women, there's always the threat of being assaulted.” (Midwifery student, FGD, Mogadishu)

Students noted that their parents expressed concerns about their children doing night shifts and having to make regular check-ups to ensure they are in the facility. Despite the safety concerns, students showed resilience and adaptability within their environment. Many participants indicated that they have grown up in this environment and adapted to the security risks.

Studying in our country can be challenging. There's a chance that when you step out the door, you will hear a bomb go off close to you, or the roads will end up being closed and you will have to walk the rest of the day. If you leave early, you might encounter a traffic jam or have to disembark from the bus and walk to class. That's the worst. But you have to look towards your goals; there's no winning anything without struggle. You need to be strong-willed and believe you can overcome this.” (Midwifery student, FGD, Mogadishu)

Despite receiving job offers, the majority of graduate students expressed their inability to work in remote areas and districts due to security concerns. Parents played a significant role in deciding the location where their daughters will work, preferring their children to work in areas where they have relatives who could provide support and a sense of security. It is also worth mentioning that many respondents reported having obtained positions in remote areas, underscoring the high demand for midwives in underserved regions.

“I received a job offer in another region, but my parents rejected that offer because my relatives do not live there, and I don't know anybody there, so I could not risk going to a place that is not secure for me.” (Recent graduate, FGD, Mogadishu)

### Perception of preparedness

The majority of midwifery graduates felt highly prepared for their roles as midwives, from admitting pregnant women to the health center until delivery, attributing their readiness to their education and practical experiences acquired from the university and on clinical practice sites. They also mentioned that they feel well prepared to provide antenatal care, postnatal care, and normal delivery, but claimed there is a gap in ultrasound, abortion care, and neonatal resuscitation.

“I can assist a mother with a normal delivery, and I spent a year practicing outside of the university to learn this and other practical skills. However, there may be some gaps in our skills, such as in the neonatal ward and C-section ward, among others.” (Recent graduate, FGD, Mogadishu)

Graduates had strong confidence in handling non-complicated childbirth cases and mentioned referring patients with complications to appropriate health care facilities, which shows that graduates are aware of their scope of practice.

‘’I am ready to work as a midwife, but if I see abnormal situations, I can refer them to the nearest hospital. I have confidence in myself and feel very prepared.’’ (Recent graduate, FGD, Mogadishu)

Graduates from both private and community schools believed that they were ready to serve as qualified midwives; however, those in community schools felt more confident because of the practical sessions they got from their schools, while those from private schools reported needing extra field practice. As the participants stated, community midwives start their training at the end of every semester, while private schools had only to practice the last year of school.

“Yes, the university gave us the theories and practices, and we are satisfied; we took practical sessions for two semesters, and the students should seek extra field practice.” (Recent graduate, FGD, Mogadishu)

“"The university equipped us with essential skills and practical experience, ensuring three months of intensive practice every semester. The high demand for our graduates confirms the quality of our training.” (Recent graduate, FGD, Galgaduud)

## Discussion

Our study found evidence for strong motivation among students and early career midwives embarking on a career in midwifery, despite formidable challenges including deep-seated mistrust of young midwives, preference for TBAs, restrictive gender norms that curtail women's workforce participation, and general devaluation and low compensation. Most participants reported intrinsic motivators such as an inspiration to provide care for mothers and children and a desire to reduce the high maternal mortality ratios, while few, if any, reported high status, money, or other extrinsic motivators when asked about why they chose midwifery. The important role played by intrinsic as opposed to extrinsic motivation among midwives is consistent with various studies conducted in other settings. For example, in Papua New Guinea, midwives were motivated by the desire to be part of a team that addresses the high maternal mortality in the country and the commitment to serving women and children (11). One study conducted in the Congo revealed that, despite facing poor working conditions and low professional status, the participants saw their profession as a calling, with the desire to save the lives of newborns and mothers serving as a powerful motivator (12). In addition to the noble goal of saving the mother newborn dyad, our study sheds light on the role TBAs have played in this context. While the community clearly favors TBAs, many participants opt to enter the workforce due to the mismanagement and poor quality care they provide to their relatives. According to a study conducted in Mogadishu, TBAs are still the first choice for pregnant women in Somalia because they are accessible in the community, pay less than private hospitals, and patients are more familiar with them because they are community members and can sometimes offer care at home (13).

Although salary, professional status, and recognition were seldom mentioned as motivating factors for joining the profession, studies have shown that these factors are essential for the retention of midwives. In Bhutan, a study assessing the factors influencing the retention of midwives working in rural areas found that factors like working and living conditions, career-related opportunities, and financial incentives such as salary, housing allowance, daily and travel allowance, and rural and altitude allowance strongly influenced the retention of midwives (14). Another study conducted in South Sudan revealed that low salaries, insufficient non-financial incentives, inadequate training, and inadequate supervision demotivated healthcare workers, leading to poor performance and high turnover (15). While financial incentives may not be crucial for attracting midwives to join the profession, they are essential for retaining them. Efforts should focus on ensuring midwives receive adequate compensation through competitive salaries as well as additional financial incentives like housing and deployment allowances.

Community perceptions of midwives in Somalia are influenced by a complex confluence of factors, including social and gender norms and traditional beliefs and preferences. Participants expressed concerns about the preference for traditional birth attendants (TBAs), who were perceived as more experienced due to their older age. In contrast, early-career midwives, who are generally younger, were viewed as less experienced in delivering babies. This pressure not only hinders the completion of their studies but also impedes their employment prospects. Another study reveals a common misconception in Somalia that women should prioritize housework over education, implying that investing time and money in girls' education is a waste (16).

Young midwives are often overlooked and dismissed as having less experience than TBAs and are thus viewed with mistrust by the community. This is by no means exclusive to the context of Somalia. In Indonesia, young village midwives are perceived as too young, untrustworthy, and not accepted by women and their families (17). Similarly, in Pakistan, TBAs wielded strong influence in the community due to their long-standing role in child delivery. However, at times, they misled the community about the role of midwives, perceiving them as competitors (18). The community, in turn, viewed community midwives as young and lacking experience. Integrating TBAs into the formal health system can be a promising strategy to both overcome the mistrust community members feel towards midwives while also leveraging the embeddedness of traditional birth attendants in the community. Evidence has shown that engaging TBAs in the health system can increase the uptake of health services and avert adverse maternal and neonatal outcomes (19).

Graduates believed they were well prepared to manage uncomplicated labor, which is encouraging. However, they identified gaps in dealing with abortion and neonatal resuscitation, indicating the need for longer clinical training periods. Most students in our study do not get enough opportunities to practice while in school, as most of the time is consumed by theoretical classes, leaving the last semester for practice. Indeed, a study conducted in Papua New Guinea exploring the education, employment, and practice of midwifery has found that more than 90% of graduates assessed were competent in providing basic midwifery skills; however, only 60% felt confident and competent in providing midwifery-specific or advanced skills as per International Confederation of Midwives guidelines (20).

Our study has some limitations. Our analysis draws on five focus group discussions with midwifery students and graduates in selected midwifery schools in Mogadishu and Galgduud, and thus experiences may not reflect those of midwives in other locations in the country that may be differently affected by violent insecurity. Despite conducting only five FGDs, we believe that saturation was achieved, as common views were consistently expressed across FGDs in relation to the study themes. Another limitation of our data is that it captures a specific point in time, focusing on the insights and experiences of midwifery students and fresh graduates. These experiences may evolve as these individuals enter the profession and gain more practice in the community. Our parent study, which will follow these participants over time, will therefore shed light on some of these changes. While we acknowledge the valuable insights provided by the emic perspective of our local researchers, their familiarity with the context may introduce potential bias. Additionally, the presence of male note-takers in a predominantly female setting could have further influenced responses. Another concern is the possibility of social desirability bias, as participants may have been hesitant to express negative opinions about the program due to fear of retaliation from their school administrators. However, this risk was mitigated by our strict confidentiality protocols, which were explained to the participants, as well as the fact that all FGDs were conducted in a neutral location away from the school, ensuring both auditory and visual privacy. Finally, despite the fact that our data collection, translation, and analysis were conducted by the first two authors, who are fluent in Somali and English, some nuance may have been lost in the translation from Somali to English.

Our study is strengthened by the positionality of the first two authors, who are from Somalia, live in Somalia, and are both trained as health workers in the country (HA is a medical doctor and AM is a midwife). Their familiarity with the Somali context likely facilitated interaction with participants and enriched the analysis and interpretation of the data. Other strengths of the study include the large number of schools from which participants were recruited, which resulted in the inclusion of a diverse set of experiences and views, as well as the collaborative nature of analysis, which took place during an in-person workshop attended by the first three authors and the senior author at Johns Hopkins.

## Conclusion

Newly graduated midwives and students face challenges that have direct implications for the health outcomes of mothers and infants in conflict-affected settings. Inadequate training leads to midwives incapable of offering appropriate care during delivery and emergencies, adding to the higher maternal and neonatal mortality rates. Additionally, security risks and restrictive gender norms might be other factors causing many mothers not to get skilled care either in pregnancy or during delivery, since midwives are not available in those areas or are at times not allowed to go to those places by their family. These challenges lead to delayed interventions, untreated complications, and reduced quality of care.

These recurring challenges highlight the urgent need for holistic responses; we recommend policymakers, governments, academic institutions, and stakeholders take further steps in improving the current situation by revising the midwifery curriculum to include extended periods of practical training in collaboration with hospitals and clinics, establishing a uniform standard for practical training across all midwifery schools, followed by periodic assessment. We suggest integrating traditional birth attendants into the healthcare system, training them, and working with qualified midwives to improve community perception about midwives. This will bolster community trust in young midwives in service and promote facility-based care.

## Data Availability

The raw data supporting the conclusions of this article will be made available by the authors, without undue reservation.

## References

[1] “Midwifery education and care.”. Available: Available online at: https://www.who.int/teams/maternal-newborn-child-adolescent-health-and-ageing/maternal-health/midwifery (Accessed: October 12, 2024).

[2] “The State of the World’s Midwifery 2021.”. Available: Available online at: https://www.unfpa.org/publications/sowmy-2021 (Accessed: July 04, 2024).

[3] Moncrieff G Downe S Maxwell M Cheyne H. Mapping factors that may influence attrition and retention of midwives: a scoping review protocol. BMJ Open. (2023) 13(10):e076686. 10.1136/bmjopen-2023-076686 37865412 PMC10603492

[4] Parry N Catling C Cummins A. Early career midwives’ job satisfaction, career goals and intention to leave midwifery: a scoping review. Women Birth. (2024) 37(1):98–105. 10.1016/j.wombi.2023.09.007 37827892

[5] UNFPA Somalia. Somalia’s maternal health workforce faces a shortage of midwives willing to relocate. (2021). Available online at: https://somalia.unfpa.org/en/news/somalias-maternal-health-workforce-faces-shortage-midwives-willing-relocate (cited 2024 June 19).

[6] Capper TS Haynes K Williamson M. How do new midwives’ early workforce experiences influence their career plans? An integrative review of the literature. Nurse Educ Pract. (2023) 70:103689. 10.1016/j.nepr.2023.103689 37393687

[7] Warsame AA. Somalia’s Healthcare System: A Baseline Study & Human Capital Development Strategy. The Heritage Institute for Policy Studies and City University of Mogadishu. (2020).Available online at: http://www.heritageinstitute.org/wp-content/uploads/2020/05/Somalia-Healthcare-System-A-Baseline-Study-and-Human-Capital-Development-Strategy.pdf (Accessed June 19, 2024).

[8] Sorbye I Leigh B. Reproductive Health: National Strategy & Action Plan 2010–2015. Somalia: UNFPA, WHO, UNICEF (2009).

[9] Maregn RT Bourret K Egal JA Esse A Mattison C Klingberg-Allvin M. Qualitative study of the roles of midwives in the provision of sexual and reproductive healthcare services in the Somaliland health system. BMJ Open. (2023) 13(3):e067315. 10.1136/bmjopen-2022-067315 36921954 PMC10030797

[10] Blomgren J Gabrielsson S Erlandsson K Wagoro MCA Namutebi M Chimala E Maternal health leaders’ perceptions of barriers to midwife-led care in Ethiopia, Kenya, Malawi, Somalia, and Uganda. Midwifery. (2023) 124:103734. 10.1016/j.midw.2023.103734 37269678

[11] Moores A Puawe P Buasi N West F Samor MK Joseph N Education, employment and practice: midwifery graduates in Papua New Guinea. Midwifery. (2016) 41:22–9. 10.1016/j.midw.2016.07.006 27498185

[12] Bogren M Grahn M Kaboru BB Berg M. Midwives’ challenges and factors that motivate them to remain in their workplace in the Democratic Republic of Congo—an interview study. Hum Resour Health. (2020) 18(1):65. 10.1186/s12960-020-00510-x 32943067 PMC7499901

[13] Mohamed AA Bocher T Magan M Omar A Mutai O Mohamoud SA Experiences from the field: a qualitative study exploring barriers to maternal and child health service utilization in IDP settings Somalia. Int J Womens Health. (2021) 13:1147–60. 10.2147/IJWH.S330069 34858064 PMC8630365

[14] Jurmin K Jariya W. Factors influencing the retention of midwives in rural areas of Bhutan: a national cross-sectional study. Open Public Health J. (2023) 16(1):e187494452308211. 10.2174/18749445-v16-230927-2023-138

[15] Mugo NS Dibley MJ Damundu EY Alam A. Barriers faced by the health workers to deliver maternal care services and their perceptions of the factors preventing their clients from receiving the services: a qualitative study in South Sudan. Matern Child Health J. (2018) 22(11):1598–606. 10.1007/s10995-018-2555-5 29956127

[16] SIDRA, Understanding the barriers to girlś and womeńs access to higher education in Puntland, Somalia A video and blogging project, Somali Institute for Development and Research Analysis. Garowe, Somalia. Available online at: https://coilink.org/20.500.12592/fnj2snon04Nov2024.COI:20.500.12592/fnj2sn (Accessed June 19, 2024).

[17] D’Ambruoso L Achadi E Adisasmita A Izati Y Makowiecka K Hussein J. Assessing quality of care provided by Indonesian village midwives with a confidential enquiry. Midwifery. (2009) 25(5):528–39. 10.1016/j.midw.2007.08.008 18215447

[18] Ahmed J Ur Rehman S Shahab M. Community midwives’ acceptability in their communities: a qualitative study from two provinces of Pakistan. Midwifery. (2017) 47:53–9. 10.1016/j.midw.2017.02.005 28242494

[19] Byrne A Caulfield T Onyo P Nyagero J Morgan A Nduba J Community and provider perceptions of traditional and skilled birth attendants providing maternal health care for pastoralist communities in Kenya: a qualitative study. BMC Pregnancy Childbirth. (2016) 16(1):43. 10.1186/s12884-016-0828-9 26931132 PMC4774132

[20] Moores A West F Puawe P Buasi N Homer CSE. Developing ’super’ midwives—motivation to become a midwife in Papua New Guinea. Women Birth. (2015) 28:S24. 10.1016/j.wombi.2015.07.083

